# Molecular clocks ticking in the court room

**DOI:** 10.1186/2041-2223-5-4

**Published:** 2014-02-28

**Authors:** Antti Sajantila

**Affiliations:** 1 Department of Forensic Medicine, Hjelt Institute, University of Helsinki, P.O.Box 40, 00014 Helsinki, Finland; 2 Institute of Applied Genetics, Department of Molecular and Medical Genetics, University of North Texas Health Science Center, 3500 Camp Bowie Blvd, Fort Worth, Texas US 76107, USA

## 

Molecular phylogenies have been used in forensic molecular epidemiological investigations of transmission events of human immunodeficiency virus (HIV) in dental and medical practices and in rape cases [1-7]. Some of these investigations have resulted in criminal charges [4-7] and typically have involved only a few transmission events in a short period of time.

Gonzáles-Candelas *et al.* report in *BMC Biology*[8] a case of an anesthetist from Valencia, Spain, who was convicted of professional malpractice by infecting over 270 of his patients with hepatitis C virus (HCV). This case differs from most other viral infection cases in that the origin of transmissions date back approximately 25 years and it involved a large number of patients. Greater than 300 candidates, who had undergone a minor surgery in one of two hospitals, were tested, and for the first time a molecular clock was used to estimate the date of various transmission events.

HCV is a single-stranded RNA virus and member of *Flaviviridae* family. It is transmitted by blood-to-blood contact, e.g. in intravenous drug use or unprotected sexual intercourse, but also during medical procedures under poor-quality conditions (e.g. non-screened blood transfusion and re-use of syringes or needles). Up to 20% of infected individuals develop severe medical complications, including liver cancer, cirrhosis with a consequent liver failure. An HCV infection is extremely devastating to an infected individual and an epidemic is costly for the society.

As the first cases were detected in 1998, and an epidemiological investigation that there was a large outbreak with possibly hundreds of patients infected, public health officials suspected that an anesthetist, working in a public and private hospital, was the likely source of the HCV transmissions. The epidemiologists investigated data of over 66,000 individuals and common risk factors for infection in surgery, e.g., surgeon, surgery room, type of surgery, anesthesiologist, and type of anesthesia. The only significant factor (adjusted Odds Ratio 28.5, 95% CI 9.83 to 82.59) was this anesthetist. This observation was supported by the fact that of the initial 197 cases considered in the outbreak, 184 had been anesthetized by the suspected individual.

Since the suspected epidemic involved an exceedingly large number of patients and the overall period or transmission events were unknown, the authorities, including the judge, tasked experts in evolutionary biology and viral epidemiology to address the following questions: 1) was the suspect the source responsible for the outbreak?; 2) Could they ascertain which patients had been infected from a common source and thus could be included in the outbreak?; 3) Alternatively, which patients could have been infected from other sources?; 4) Could they exclude these alternative sources or the existence of different but simultaneous outbreaks?; 5) Could they determine the duration of the outbreak?; 6) Could they date the time of infection for each patient in the outbreak?; and 7) Could they determine the date of infection of the anesthetist?

Gonzáles-Candelas *et al*. [8] tackled the questions by sequencing 229-nucleotides (nts) of a conservative non-structural NS5B gene by reverse transcription of RNA to DNA, followed by Sanger sequencing of the DNA in all patients, the anesthetist, and a number of control samples.

The difficulty with genetic analysis of HCV is, like HIV, it is a very fast-evolving virus, and thus, even individuals harboring the virus with the same origin will have differences in their viral RNA sequences. However, those sequences from the same source are likely to be more similar than those of epidemiologically unassociated patients. Therefore, phylogenetic approaches could prove informative, as was practiced in some previous cases analyzing HIV sequences [1-6]. Gonzáles-Candelas *et al*. [8] typed patients positive for the 320 HCV-1a and 290 HCV-1b strains and 44 HCV-1a positive patients serving as geographically local controls. The HCV from the anesthetist was genotypically consistent with HCV-1a, and a dual (HCV-1A and -1b) origin was excluded. The authors then analyzed only the HCV-1a positive patients, and compared them with the controls. The sequence differences along the targeted 229 nts of the NS5B gene among the patients vs among the controls compared to the anesthetist were clear: there were 0–19 differences among the patients of which half had exactly the same sequence as the anesthetist and three out of four had two or less differences. The challenge of determining the direction and timing of the infection still remained, since phylogenetic trees reconstructed from the relatively short and slowly evolving NS5B gene did not have sufficient resolving power to separate the branches of the patients and local controls.

Analysis then was performed on a faster evolving 406-nt region in the structural envelope coding E1-E2 region. The samples from the anesthetist were taken on 12^th^ Feb 1998, and no further sampling was possible under Spanish law. Altogether over 4000 cloned sequences were used for the phylogenetic analysis, and 134 of them represented the anesthetist with 28 different haplotypes, which clustered in two groups. Neighbor-joining (NJ) and maximum-likelihood (ML) based phylogenetic trees showed highly supported internal branching (100% and 96% bootstraps, respectively). These trees were used to define which patients were included in the outbreak. This analysis divided the group into 274 patients considered associated with the outbreak (victims), 47 patients initially thought to be in the outbreak that were excluded, and 47 controls. In order to assess the support for this grouping the authors calculated the likelihood ratio (LR) for alternating hypotheses that the sequences of the patients group with the outbreak vs they group with the controls. The LRs tended to favor the hypothesis that they fall within the outbreak group and ranged from 1.051 to 6.622 × 10^95^. Similarly, for those excluded from the outbreak the LRs ranged from 1.330 to 4.408 × 10^84^ supporting the alternate hypothesis. Both of the figures provided a very strong support for the conclusions.

For estimating the timing of the transmission events Gonzáles-Candelas *et al.*[8] used a Bayesian method implemented in the program BEAST (Bayesian Evolutionary Analysis by Sampling Trees). For each of the 274 patient they used the E1-E2 region sequences to estimate the time to the most recent common ancestor (MRCA). For the analysis they incorporated the dates of the sampling, and the dates of the infection of 24 patients, who visited the anesthetist only once. Sequences from these 24 patients were used to calibrate molecular clock estimates for the MRCA. The authors concluded that the estimated transmission events of the patients was between Jan 1987 and April 1998, compared to the one obtained for the anesthetist August 1984 to October 1991). In the court these data were compared with those obtained from the non-molecular investigation (documents and testimonies), and in two out of three cases the molecular data estimates were congruent with other data.

The analysis in this case was systematic and rigorous. However, it is important to note that the data presented by Gonzáles-Candelas *et al.*[8], like in many other DNA investigations presented in the court, were not used to prove if the anesthetist was guilty, but rather in concert with other evidence. Even though only a piece of the puzzle, analyses, such as carried out by Gonzáles-Candelas *et al*., will likely be used increasingly to assist epidemiologic and criminal (also civil) investigations.

## Competing interests

The authors declare that they have no competing interests.
